# Vitamin A deficiency and associated risk factors in children aged 12–59 months living in poorest municipalities in the South Region of Brazil

**DOI:** 10.1017/S1368980022000325

**Published:** 2022-02-07

**Authors:** Camila Dallazen, Daniela Cardoso Tietzmann, Sara Araújo da Silva, Eduardo Augusto Fernandes Nilson, Vivian Siqueira Santos Gonçalves, Regina Maria Ferreira Lang, Sandra Patricia Crispim, Júlia Dubois Moreira, Solange Cristina Garcia, Márcia Regina Vítolo

**Affiliations:** 1 Nutrition Research Group (NUPEN), Federal University of Health Sciences of Porto Alegre, Sarmento Leite 245, Porto Alegre 90050-170, Brazil; 2 Brazilian Ministry of Health, Brasília, Brazil; 3 University of Brasília, Brasília, Brazil; 4 Federal University of Paraná, Curitiba, Brazil; 5 Federal University of Santa Catarina, Florianópolis, Brazil; 6 Federal University of Rio Grande do Sul, Porto Alegre, Brazil

**Keywords:** Vitamin A deficiency, Risk factors, Child, Cross-sectional studies

## Abstract

**Objective:**

To estimate the prevalence of vitamin A deficiency (VAD) in children and associated risk factors.

**Design:**

Analysis of data from a cross-sectional multicentre study performed in the primary care units of the municipalities from January to June 2015. The children’s legal guardians answered a socio-economic questionnaire, and the children’s blood samples were obtained by venipuncture. Plasma retinol was determined by HPLC. Plasma retinol values of <0·70 μmol/l were considered VDA. Poisson multiple regression with robust variance was used. Values of *P* < 0·05 were considered significant. The data were analysed in the SPSS software, 21.0.

**Setting:**

Forty-eight poorest municipalities in the South Region of Brazil.

**Participants:**

Children (*n* 1503) aged 12–59 months.

**Results:**

The prevalence of VAD in the sample was 1·9 % (95 % CI (0·5, 6·8)). The following risk factors were associated with the outcome in the final explanatory model: family received Bolsa Familia program benefits (PR = 3·19; 95 % CI (1·69, 6·02)), child was not being breastfed (PR = 5·22; 95 % CI (1·68, 16·18)) and stunting (PR = 4·75; 95 % CI (2·10, 10·73)).

**Conclusions:**

VAD did not represent a public health problem for children living in socio-economically vulnerable municipalities in the South Region of Brazil, suggesting a new panorama of this nutritional deficiency even in regions of low socio-economic conditions in these three states. Thus, in view of the current nutritional transition scenario, it is necessary to continuously monitor and improve public policies related to vitamin A supplementation in the country.

Vitamin A deficiency (VAD) is a major public health problem that affects approximately 190 million pre-school children worldwide^(1)^. Despite the existing strategies in Brazil to prevent childhood nutritional deficiencies in Brazil and advances have been made in the area, VAD affected 17·4 % of children under 5 years of age, according to data from the 2006 National Demographic and Health Survey (NDHS). The highest and lowest prevalences were identified in the Southeast (21·6 %) and South regions (9·9 %), respectively^(2)^.

The impacts of VAD on children include ophthalmological manifestations such as risk of blindness, increased morbidity due to infectious diseases (e.g. diarrhoea and measles) and increased mortality among children under-5 years of age^(3)^. The greater vulnerability of pre-school children to VAD is related to their rapid growth and development in this life stage and a consequent increase in their vitamin A requirements, which is often not supplied due to insufficient intake^(3)^. Children in this age group are also more susceptible to diseases (mainly gastrointestinal and respiratory infections) that reduce absorption and increase the biological utilisation and excretion of this vitamin, thus leading to the development of VAD^(4)^.

VAD is a multifactorial problem and has among its main determinants socio-economic status, especially income. The low family purchasing power can generate a situation of food and nutritional insecurity, reflecting on a low-quality diet^(5,6)^ and, consequently, the greater risk of nutritional deficiencies, especially in more vulnerable groups such as children^(7)^. In this context, considering that an increase in the income of low-income families may have a positive impact on access and quality of food, the federal government has been implementing, since 2003, conditional cash transfer programmes such as the Bolsa Familia Program (BFP). This program became the largest social program to target the poorest households in the country, reaffirming the position and role of the State in tackling inequality, poverty and social exclusion. One of the objectives of the PBF is to promote the independence of the beneficiary families. It is expected that the beneficiaries have improved living and health conditions and are able to support themselves without government intervention^(8)^. Despite some advances in the country’s social inclusion policies during this period, food and nutritional insecurity^(9,10)^ and micronutrient deficiencies^(11)^ are still a public health problem with wide inter- and intraregional variations^(9–11)^.

The strategic measure that has been implemented in Brazil since 2005 consists of distributing megadoses of vitamin A to children aged 6–59 months through the National Vitamin A Supplementation Program (NVASP)^(12)^. The strategy is in line with the recommendations of the WHO that recommends vitamin A supplementation with a megadose of 100 000 international units (IU) for infants aged 6–11 months and 200 000 IU at least twice a year for children aged 12–59 months who live in places where VAD is considered a public health problem^(3)^. At the same time, actions are recommended to promote exclusive breast-feeding up to the sixth month of life and complemented up to 2 years of age and beyond with adequate and healthy food, ensuring the consumption of vitamin A-rich foods by the population^(12)^. The National Vitamin A Supplementation Program is part of the Brazil Caring Action, which is part of the Brazil Without Poverty Plan (BWPP), ensuring the vitamin’s availability to all children in this age group in the North and Northeast regions as well as the municipalities of the Midwest, Southeast and South regions that participate in the BWPP^(13)^.

The South Region of Brazil had the lowest prevalence of VAD in children according to data from the last national survey^(2)^, and there is a lack of recent studies to evaluate VAD trends in the South Region of Brazil (especially in areas of low socio-economic status). The current study aimed to estimate the prevalence of and risk factors associated with VAD in children aged 12 to 59 months living in poorest municipalities in the South Region of Brazil.

## Methods

The current study consists of an analysis of data from a cross-sectional multicentre study, the main objective of which was to assess the prevalence of VAD and anaemia in children from 12 to 59 months of age living in poorest municipalities in the South Region of Brazil. Data were collected from January to June 2015 in a sample of forty-eight municipalities included in the BWPP, located and distributed equally among the three states comprising the South Region of Brazil: Paraná, Santa Catarina and Rio Grande do Sul. The South Region is the smallest of Brazil’s five regions, with a population of about 30 million inhabitants. The region has the lowest infant mortality rate, the highest literacy rate and the highest life expectancy at birth among all of the country’s regions. However, in the year 2015, 9·6 % of the population in the South were classified as low-income (poor or extremely poor), i.e. surviving on a per capita monthly income of one half the minimum wage (approximately USD 130 per capita/month)^(14)^. The BWPP’s objectives were to (1) raise the per capita family income of people living in extreme poverty; (2) increase access to public services for people living in extreme poverty and (3) provide people living in extreme poverty with access to employment and income opportunities through productive inclusion actions. In the year 2014 (during the study’s planning phase), the BWPP in the South of Brazil included 255 municipalities: ninety four in Paraná State, fifty nine in Santa Catarina State and 102 in Rio Grande do Sul State^(15)^.

Details of the sampling process were previously published^(16)^. The sample size was calculated based on the main study (prevalence of anaemia and hypovitaminosis A in children aged 12–59 months living in the poorest municipalities in the South Region of Brazil) considering the 21·5 % prevalence of anaemia in children under 5 years of age in the South Region^(2)^, 95 % confidence level, infinite population and design effect (*deff*) of 1·5. In each of the three states, the primary sampling unit (PSU) was defined as the set of municipalities included in the BWPP in the year 2014 and with zero coverage in the NVASP in the year 2014. The criterion of zero coverage in the NVASP was considered to not interfere with the results of the study. The NVASP coverage information was obtained through data made available by the Ministry of Health. The PSU in the state of Paraná presented forty-eight eligible municipalities, Santa Catarina presented forty two and Rio Grande do Sul presented thirty nine.

For the sampling process, the selection of at least one-third of the eligible municipalities in each PSU was determined in order to reduce the sample effect. Considering that the largest PSU contained forty-eight eligible municipalities, sixteen municipalities were selected in each PSU. For the selection, the eligible municipalities of each PSU were included in a spreadsheet and sorted in ascending order according to the estimated number of children aged 12 to 59 months. Then, the sampling interval was calculated by dividing the number of municipalities in each PSU by the number of necessary municipalities. A number less than or equal to the interval was drawn to determine the first municipality to be selected in each PSU. Thus, the estimated sample size for each PSU was of 389 children, which was increased by 25 % to account for potential losses and rounded, resulting in 500 children per PSU and a total sample size of 1500 children aged 12–59 months^(16)^. Children aged 6–11 months were not included due to unavailability of data covering in NVASP in this age group.

Due to issues of logistic complexity and difficulties in the prior identification of eligible children because of the lack of current registries in the participating municipalities, the selection of participants for the study used a convenience sampling. The identification and recruitment of children were done by the reference primary healthcare teams in each municipality, identified by the local administration. The Municipal Health Departments were contacted for this purpose, after their agreement to participate in the study, and they were asked to recommend one or more primary care units (PCU) that could cooperate with the study, considering the availability of healthcare professionals for identifying children in the target age bracket and an appropriate place to house the data collection (spacious and that guaranteed biosafety). After definition of the PCU, each health team was contacted by telephone. During the first contact with the health team, the study’s objectives were explained, and the PCU was asked to recommend a staff member that could be in charge of the local coordination of the strategy to publicise the study among the families with children 12 to 59 months of age and to arrange the place for the data collection. During the second telephone contact, the local health professional at each PCU received instructions for identifying eligible children for the study, and the dates were scheduled for the data collection. Each local health professional also received a manual online containing instructions for the identification of eligible children. In all the PCU, the invitation to parents of children to participate in the study was done through the community health workers, when available, and through announcements in local communications media such as community radio stations, churches, posters in the waiting rooms of the PCU and during appointments with health professionals at the PCU. All children whose children’s parents/legal guardians responded to recruitment entered the study until the desired number was reached^(16)^.

Children were considered ineligible if they had undergone a blood or blood product transfusion, were receiving an immunosuppressive or cortico-therapeutic therapy, had a chronic disease, had HIV or a serious infectious process (such as pneumonia, bacterial meningitis, measles, rubella and chickenpox), had a congenital malformation and/or had been hospitalised for diarrhea in the previous month^(16)^. All of these criteria were considered because inflammation can reduce plasma retinol concentration^(17)^.

Data collection was done by two nutritionists hired for the study in each state, trained in the study protocol, on 2 d previously scheduled in each municipality. Data were collected through face-to-face interviews with the children’s parents/legal guardians at a PCU physical location in each municipality using a pre-coded structured questionnaire to obtain socio-demographic information^(16)^. Information on birth weight and previous supplementation with multivitamins and vitamin A megadoses was collected using the child’s health record as the primary information source while also checking with the parent/guardian. Although the sampling process was performed considering only the municipalities not covered by the NVASP in 2014, information on the use of vitamin A megadose supplementation was collected due to the possible participation of selected municipalities in the programme in the period between the study’s planning and data collection stages.

The children’s weight and length/height were measured using standardised procedures^(18)^. Weight was measured with a Marte® (São Paulo) electronic scale with a capacity of 150 kg and sensitivity of 100 g. Children under 24 months of age were weighed and measured completely naked (without diapers) in the presence of his/her mother/guardian. The children were weighed while sitting in their mother’s lap and the mother’s weight was later deducted. Children aged 24–59 months were weighed while standing barefoot in light clothing. Height/length was measured with a portable stadiometer (Alturaexata®, Belo Horizonte) with a one-metre extension and precision to 0·1 cm. Children under 24 months of age were measured in the dorsal decubitus position and older children were measured while standing and barefoot. Measurements were recorded in centimetres. Nutritional status was assessed using the BMI for age (BMI/A) and height for age (H/A), expressed as mean *Z*-scores. The WHO’s growth curves were adopted using the Anthro program (http://www.who.int/childgrowth/software/en/). BMI/A values with *Z*-scores of >1 were classified as overweight, including children who were classified as being at risk of overweight (*Z*-score > *z* + 1 and ≤ *z* + 2), overweight (*Z*-score > *z* + 2 and ≤ *z* + 3) and obese (*Z*-score > *z* + 3). Stunting was defined as height-for-age *Z*-score < −2 based on the WHO Child Growth Standards^(19)^.

The blood samples were collected by nurses or nursing technicians who were duly trained for the study. Some nurses or nursing technicians had a link to their respective health teams while others did not. The blood samples were obtained by peripheral venipuncture. Aliquots of six to eight millilitres of blood were collected using disposable needles and syringes and all were placed in EDTA tubes. All blood samples were taken in the morning without requiring prior fasting and were placed in tubes under soft lighting. The samples were centrifuged at 1500 rpm for 10 min, and two aliquots of plasma were placed in amber Eppendorf tubes. The samples from the municipalities were stored and transported refrigerated to the analysis laboratory. They were then stored in a freezer at -80 °C until they were analysed.

Plasma retinol was quantified using the method for the simultaneous quantification of lycopene, *β*-carotene, retinol and *α*-tocopherol by HPLC with a Shimadzu® fluorescence detection with isocratic according to the technique proposed by Charão *et al.* ^(20)^ at the Toxicology Laboratory of the Federal University of Rio Grande do Sul. For this purpose, 90 μl of plasma was mixed with 450 μl of ethanol: n-butanol solution (50:50, v/v) solution containing 5 mg of hydroxytoluenebutylated/ml. The solution was homogenised for 10 s and then kept in ice for 5 min. Subsequently, it was homogenised again in the vortex for 10 s and then centrifuged at 2700 *g* for 3 min in a refrigerated Thermo Scientific® centrifuge. After that, 400 μl of the clear supernatant was transferred to vials for retinol quantification. A diagnosis of VAD was determined when plasma retinol values were <0·70 μmol/l^(21)^.

Quality control for the collected data was systematically performed throughout the data collection stage. All data were double-entered and validated in the Epi-Data 3.2 program (Epidata Association, Odense, Denmark). Data analysis was performed in the SPSS 21.0 statistical program (IBM Corp.). The multicollinearity test was performed using the variance inflation factor with a cut-off point of >10. The test showed an absence of multicollinearity among the independent variables studied.

Sample characteristics were described by relative frequency (%) and respective 95 % CI for categorical variables and mean and SD for continuous variables. VAD prevalence was analysed according to its association with independent variables (i.e. socio-demographic and maternal factors, factors related to health care access and factors related to the child). The bivariate analyses were performed using Poisson regression to obtain the point estimates and intervals of the prevalence ratios and to test the associations of the independent variables with the outcome.

**Fig. 1:**
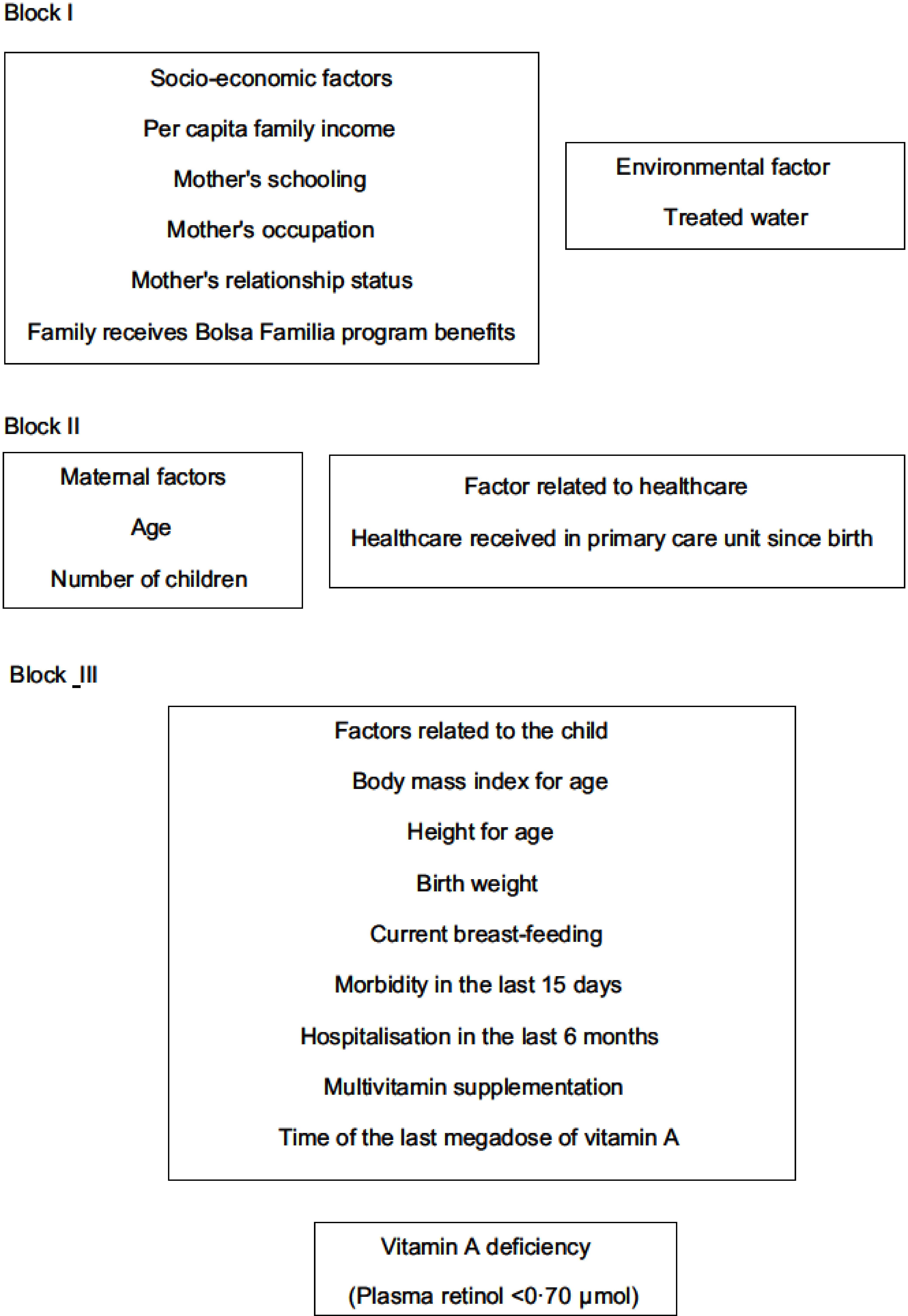
Hierarchical causal model of the process to determine vitamin A deficiency in children aged 12 to 59 months, South Region, Brazil, 2015

The strategy outlined for the multiple analysis followed a previously proposed hierarchical model to determine VAD that is theoretically based on the logical relationships between the event and its determining factors. The independent variables were selected and classified based on the literature^(1,4,21–26)^ and grouped into three blocks (Fig. 1)^(27)^. The first block was composed of socio-economic and environmental factors: per capita family income (≤ BRL 394; > BRL 394), mother’s schooling (≤8 years; > 8 years), mother’s occupation (homemaker; other), mother’s relationship status (with partner; without partner), family receives BFP benefits (yes; no) and access to treated water (no; yes). The per capita family income was classified based on the Brazilian government cut-off point criteria to identify low-income families. The homemaker was considered the woman who works exclusively for her own family (unpaid job). Treated water was considered to be water subjected to physical treatment, chemical treatment or a combination of these processes. The second block included variables related to maternal factors and healthcare: age (<20 years old; ≥20 years old), number of children (1; >1) and health care received in PCU since birth (no; yes). Health care received in PCU since birth was considered childcare such as monitoring growth and development, immunisation and others. The third block’s level consisted of factors related to the child: BMI/A (≤ + 1 *Z*-score; > + 1 *Z*-score), H/A (< - 2 *Z*-score; ≥ - 2 *Z*-score), birth weight (<2500 g; ≥2500 g), current breast-feeding (no; yes), morbidity in the last 15 d (yes; no) of hospitalisation in the last 6 months (yes; no) and multivitamin supplementation (never used; uses/has used) and time of the last megadose of vitamin A (> 6 months; ≤ 6 months). Morbidity in the last 15 d was considered if the child had any disease (respiratory, infectious, parasitic and others) in the 15 d prior to the interview.

Multiple Poisson regression with robust variance for the parameter estimates was used to obtain the adjusted estimates of the prevalence ratios, and a multivariate analysis was performed following the strategy proposed by Victora *et al.* ^(27)^ The backward method was used considering each level of the model progressively. On each level, the variable was withdrawn with a *P*-value greater than the others and with a significance of *P* ≥ 0·20, until all variables presented a value of *P* < 0·20. Variables from the second hierarchical level were then added to this model proceeding in the same way, with the progressive exclusion of this level’s variables. All three hierarchical levels were analysed this way. After completing the analysis of each of the model’s levels, the remaining variables were kept in the modelling process to fit the final model, in which the variables with *P* < 0·05 were considered significant. In all of the analyses, the sample design effect for complex samples was considered using sample weights calculated in the Complex Samples module of the SPSS 21.0 statistical program (IBM Corp.).

## Results

A total of 1567 children aged 12 to 59 months participated in the study. It was not possible to determine the plasma retinol levels of sixty-four children due to the child’s and/or guardian’s refusal to be tested or to technical factors inherent to the analysis method. Thus, 1503 children were evaluated, of which 56·6 % (95 % CI (48·4, 64·5)) were males. The mean age was 34·9 months (sd ± 13·6). Regarding socio-demographic factors, the mean per capita family income was BRL 434·52 (sd ± 439·14) (approximately USD 144 in 2015), and the mean number of residents in the household was 4·1 people (sd ± 1·3). The mean age of the mothers/legal guardians was 29·4 years (sd ± 7·7) and 8·1 % (95 % CI (4·6, 13·9)) of the mothers had 4 years of schooling or less. Approximately 96 % (95 % CI (79·7, 99·2)) of the PCU from which the children were selected had the Family Health Strategy model and 86·4 % (95 % CI (79·0, 91·5)) of the families received home visits from community health agents (data not shown in the table).

The prevalence of VAD (<0·70 μmol/l) and severe VAD (<0·35 μmol/l) in the sample was 1·9 % (95 % CI (0·5, 6·8)) and 0·5 % (95 % CI (0·1, 2·0)), respectively. The prevalence of VAD risk (<1·05 μmol/l) was 4 % (95 % CI (1·1, 14·1)), and most of the children (94 %, 95 % CI (79·9, 98·4)) presented acceptable plasma retinol levels (≥1·05 μmol/l). The median plasma retinol distribution (μmol/l) was 1·53 μmol/l and the 10th, 25th and 75th percentiles were 0·78 μmol/l, 1·16 μmol/l and 1·90 μmol/l, respectively. The mean plasma retinol concentration observed was 1·68 μmol/l (95 % CI (1·62, 1·74)). The plasma retinol concentrations showed homogeneous distributional behaviour in relation to the following variables: number of residents in the same household, age, maternal schooling, mother’s number of children, child’s sex/age, medical care received at the PCU since birth, access to treated water, multivitamin supplementation and time since the last vitamin A megadose (Table 1).

**Table 1:** Sample characterisation according to socio-demographic and maternal factors and factors related to the child and his/her plasma retinol level (μmol/l), South Region, Brazil, 2015

Variable	%	95 % CI	Retinol plasma (μmol/l)		
			Mean	95 % CI	*P*
Socio-demographic factors					
PSU (*n* 1503)					
1	34·7	32·3, 37·3	1 72	1·56, 1·88	<0·001
2	34·3	31·9, 36.	1·71	1·67, 1·75	
3	31·0	32·3, 37·3	1·24	1·05, 1·42	
Number of residents in home (*n* 1435)					
≤4 residents	55 0	41·3, 67·9	1·76	1·60, 1·92	0·079
> 4 residents	45·0	32·1, 58·7	1·60	1·56, 1·64	
Per capita family income* (*n* 1379)					
≤ BRL 394	61·8	59·1, 64·4	1·65	1·59, 1·70	0·002
> BRL 394	38·2	35·6, 40·9	1·79	1·67, 1·90	
Mother’s schooling (*n* 1450)					
≤8 years	44·8	37·9, 51·9	1·69	1·59, 1·80	0·583
> 8 years	55·2	48·1, 62·1	1·67	1·62, 1·71	
Mother’s occupation (*n* 1433)					
Homemaker	40·1	35·1, 45·1	1·56	1·47, 1·64	0·020
Other	59·9	54·9, 64·6	1·77	1·66, 1·88	
Mother’s relationship status (*n* 1472)					
With partner	19·6	16·2, 23·6	1·54	1·46, 1·61	0·011
Without partner	80·4	76·4, 83·8	1,72	1·63, 1·80	
Bolsa Familia Program benefits (*n* 1461)					
Yes	38·9	31·8, 46·6	1·50	1·42, 1·57	<0·001
No	61·1	53·4, 68·2	1·80	1·71, 1·89	
Environmental factors					
Treated water (*n* 1443)					
No	76·3	71·9, 80·3	1·69	1·62, 1·77	0·302
Yes	23·7	19·7, 28·1	1·65	1·60, 1·70	
Maternal factors					
Age (*n* 1450)					
<20 years	12·3	8·9, 16·8	1·71	1·66, 1·77	0·357
≥20 years	87·7	83·2, 91·1	1·68	1·62, 1·75	
Skin colour (*n* 1436)					
Non-white	39·8	36·4, 43·3	1·60	1·54, 1·66	<0·001
White	60,2	56·7, 63·6	1·74	1·68, 1·80	
Number of children (*n* 1457)					
>1	60·7	56·8, 64·6	1·68	1·60, 1·76	0·843
1	39·3	35·4, 43·2	1·69	1·63, 1·74	
Factors related to healthcare					
Health care received in PCU since birth (*n* 1463)					
No	3·2	0·8, 11·4	1·84	1·51, 2·17	0·304
Yes	96·8	88·6, 99·2	1·68	1·62, 1·74	
Factors related to the child					
Sex (*n* 1503)					
Female	56·6	48·4, 64·5	1·75	1·65, 1·84	0·065
Male	43·4	35·5, 51·6	1·60	1·50, 1·69	
Age (*n* 1503)					
12–23 months	51·1	32·4, 69·5	1·66	1·62, 1·69	
24–35 months	19·3	14·2, 25·7	1·63	1·57, 1·70	
36–47 months	18·9	14·2, 24·8	1·86	1·61, 2·12	
48–59 months	10·6	4·5, 22·9	1·58	1·43, 1·73	0·189
Skin colour (*n* 1460)					
Non-white	26·5	24·0, 29·2	1·57	1·52, 1·63	<0·001
White	73·5	70·8, 76·0	1 72	1·67, 1·78	
Attends childhood education school (*n* 1479)					
No	64·1	55·0, 72·4	1·71	1·66, 1·76	<0·001
Yes	35·9	27·6, 45·0	1·63	1·57, 1·69	
BMI/A (*n* 1336)					
≤ + 1 *Z*-score	70·9	67·1, 74·5	1·71	1·64, 1·77	0·001
v> + 1 *Z*-score	29·1	25·5, 32·9	1·63	1·59, 1·68	
H/A (*n* 1352)					
< - 2 *Z*-score	4·4	3·6, 5·4	2·39	1·68, 3·10	0·034
≥ - 2 *Z*-score	95·6	94·6, 96·4	1·65	1·61, 1·70	
Birth weight (*n* 1354)					
<2500 g	8·2	7·1, 9·3	1·40	1·31, 1·50	<0·001
≥2500 g	91·8	90·7, 92·9	1·71	1·64, 1·78	
Current breast-feeding (*n* 1444)					
No	76·6	67·6, 83·6	1·67	1·61, 1·73	0·001
Yes	23·4	16·4, 32·4	1 72	1·67, 1·77	
Multivitamin supplementation (*n* 1459)					
Never used	75·0	67·2, 81·5	1·69	1·63, 1·74	0·338
Uses/has used	25·0	18·5, 32·8	1·73	1·61, 1·84	
Vitamin A megadose supplementation (*n* 1462)					
No	92·3	74·5, 98·0	1·70	1·65, 1·75	0·011
Yes	7·7	2·0, 25·5	1·52	1·39, 1·64	
Time since last vitamin A megadose supplementation (*n* 487)					
> 6 months	95·9	85·3, 99·0	1·46	1·28, 1·64	0·288
≤ 6 months	4·1	1·0, 14·7	1·55	1·38, 1·73	

PSU, primary sample unit; PCU, primary care unit; BMI/A, BMI for age; H/A, height for age.

* Cut-off point established in the Ministry of Social and Agrarian Development criteria to identify and characterise low-income families (approximately USD 130 in 2015).

VAD prevalence among the independent variable categories and the crude and adjusted prevalence ratios between the outcome and the independent variables are presented in Table 2. In Block I, only the variable of the families that received Bolsa Familia program benefits reached the threshold adopted (*P* < 0·20) to enter the next stage of the analysis model. In Block II (maternal factors and factors related to health care access), none of the variables reached the selective level for the bivariate analyses. In Block III (proximal factors related to the child), height for age, current breast-feeding and time since the last vitamin A megadose presented values of *P* < 0·20. Finally, receipt of Bolsa Familia benefits, stunting and current breast-feeding remained associated with the outcome in the explanatory model in the intra- and intergroup adjusted analyses (Table 2). The probability of presenting VAD was approximately 3·2 (95 % CI (1·69, 6·02)) times higher for children whose families received Bolsa Familia program benefits. As for the factors related to the child, the children who were not being breastfed were more likely to present VAD (PR 5·22; 95 % CI (1·68, 16·18)) than those who were being breastfed. The probability of presenting VAD was 4·75 (95 % CI (2·10, 10·73)) times higher for children who presented stunting.

**Table 2:** Prevalence of factors associated with vitamin A deficiency (VAD) in children aged 12–59 months, South Region, Brazil, 2015 (*n* 1503)

Blocks/variable	PR_gross_	95 % CI	*P*	PR_adjusted_	95 % CI	*P*
Block I						
Socio-economic factors						
Per capita family income*						
≤ BRL 394	1·44	0·81, 2·54	0·195	0·96	0·48, 1·94	0·924
> BRL 394	1·00			1·00		
Mother’s schooling						
≤8 years	1·10	0·60, 2·03	0·749	1·47	0·76, 2·84	0·243
> 8 years	1·00			1·00		
Mother’s occupation						
Homemaker	1·06	0·47, 2·39	0·882	0·92	0·30, 2·81	0·894
Other	1·00		0·882	1·00		
Mother’s relationship status						
With partner	1·08	0·40, 2·92	0·867	1·17	0·34, 4·04	0·791
Without partner	1·00			1·00		
Bolsa Familia Program benefits						
Yes	2·66	1·55, 4·55	<0·001	3·19	1·69, 6·02	
No	1·00			1·00		
Environmental factor						
Treated water						
No	1·31	0·69, 2·49	0·393	−0,77	0·37, 1·58	0·471
Yes	1·00			1·00		
Block II						
Maternal factors						
Age						
<20 years	0·79	0,26, 2·42	0·681	1·58	0·52, 4·76	0·403
≥20 years	1·00			1·00		
Number of children						
>1	0·90	0·56, 1·43	0·659	1·37	0·78, 2·39	0·263
1	1·00			1·00		
Factor related to healthcare						
Health care received at PCU since birth						
No	1·15	0·13, 9·60	0·895	0·74	0·09, 6·07	0·779
Yes	1·00			1·00		
Block III						
Factors related to the child						
BMI/A						
≤ + 1 *Z*-score	0·85	0·53, 1·38	0·520	0·95	0·49, 1·83	0·881
> + 1 *Z*-score	1·00			1·00		
H/A						
< - 2 *Z*-score	4·48	2·15, 9·33	<0·001	4·75	2·10, 10·73	<0·001
≥ - 2 *Z*-score	1·00			1·00		
Birth weight						
< 2500 g	1·32	0·55, 3·19	0·516	0·78	0·27, 2·21	0·634
≥ 2500 g	1·00			1·00		
Current breast-feeding						
No	4·25	1·44, 12·58	0·007	5·22	1·68, 16·18	0·005
Yes	1·00			1·00		
Morbidity in the last 15 d						
Yes	1·06	0·56, 1·99	0·839	0·96	0·36, 2·55	0·940
No	1·00			1·00		
Hospitalisation in the last 6 months						
Yes	3·06	0·62, 15·05	0·157	0·94	0·14, 6·17	0·952
No	1·00			1·00		
Multivitamin supplementation						
Never used	0·61	0·27, 1·35	0·211	1·69	0·56, 5·09	0·336
Uses/has used	1·00			1·00		
Time since last vitamin A megadose supplementation						
> 6 months	0·25	0·04, 1·39	0·106	3·26	0·65, 16·35	0·145
≤ 6 months	1·00			1·00		

PR, prevalence ratio; PCU, primary care units; BMI/A, BMI for age; H/A, height for age.

* Cut-off point established in the Ministry of Social and Agrarian Development criteria to identify and characterise low-income families (approximately USD 130 in 2015.

## Discussion

The prevalence of VAD in children aged 12–59 months in the current study was 1·9 % and therefore did not represent a public health problem according to WHO criteria^(20)^. The risk factors independently associated with VAD in this population were the receipt of Bolsa Familia program benefits, stunting and not being breastfed.

The VAD prevalence identified in the current study is lower than that found in similar studies conducted in different regions of the country with populations of the same age, whose prevalence ranges from 16 % to 45·4 %^(22,23,28)^. Brazil’s South Region has a lower prevalence of nutritional deficiencies in children, including VAD, as shown by the National Survey of Demography and Children’s and Women’s Health, which identified a prevalence of 9·9 %, representing the lowest prevalence found among Brazil’s five regions^(2)^. Although VAD is still epidemiologically relevant in regions that share risk factors such as poverty, infectious diseases and low availability of dietary sources of vitamin A^(29)^, there is evidence that shows a gradual decrease in the occurrence of this deficiency in recent decades, especially in countries considered to be low-to-middle income^(30)^. In Brazil, there are no national estimates that provide a reliable panorama of the occurrence of VAD in children. However, improvements in child nutrition and health conditions such as decreases in mortality and height deficit coefficients, a reduction in malnutrition and increased access to health care^(31)^ may have contributed to reducing the prevalence of nutritional deficiencies, including VAD.

In the present study, the children whose families were Bolsa Familia program beneficiaries had a higher occurrence of VAD compared with those whose families were not. Previous studies have shown better nutrition and health conditions in children whose families are BFP beneficiaries compared with children whose families are eligible for the BFP but are not enrolled in the program, nevertheless. These conditions include decrease in childhood mortality, and particularly for deaths attributable to poverty-related causes such as diarrhea and malnutrition^(32)^, increased food security^(33)^, increased child development monitoring^(34)^ and improved diet quality and diversity^(35)^. Our results suggest a relationship between socio-economic marginalisation and the occurrence of deficiency diseases in childhood. Studies related to income transfer programmes and micronutrient deficiencies in Brazil’s child population were not identified in the scientific literature. Thus, it is possible that meeting the conditions of remaining in the BFP (monitoring the vaccination schedule and taking anthropometric measurements) is not sufficient for the prevention and early identification nutritional deficiencies. Other routine services such as actions of nutrition education and conducting clinical and laboratory tests could be more effective for the prevention and timely diagnosis of these deficiencies. However, these services require public investment and depend on the organisation of health services. In addition, although the BFP promotes access to basic health care, its benefits may be limited by the quality of existing services, since monitoring the programme’s conditions does not necessarily mean the insertion of comprehensive care actions for children’s health.

The greater occurrence of VAD in children whose families were BFP beneficiaries was expected since the BFP prioritises the inclusion of highly vulnerable families. This cross-sectional cut is not intended to quantify the programme’s impact on health conditions since the conditions of these beneficiaries were possibly worse than those of the other participants prior to the study. Thus, the results underscore the BFP’s importance in reducing existing inequities in the Brazilian population, which will only be possible with economic changes and social inclusion besides public investment and improving the quality of health services. These results should be interpreted with caution considering that the time at which the families received BFP benefits was not verified. Such a relationship can be investigated in future research.

In regard to factors related to the child, there was an association between VAD and stunting. Stunting (growth retardation) is an important indicator of malnutrition because it reflects the long-term cumulative effects of a poor diet and/or recurrent infections^(36)^ and is also associated with low socio-economic conditions^(37,38)^. Although there is no consensus in the literature^(39)^, the results of observational studies conducted with pre-school children in Sri Lanka^(40)^ and Uganda^(41)^ have corroborated the current study’s findings. On the other hand, a study conducted with children aged 12–72 months in Brazil showed no association between VAD and stunting^(42)^. The depth and course of nocturnal sleep is known to be a potent stimulator of growth hormone secretion^(43)^. There are indications that plasma retinol levels are correlated with the abnormal nocturnal secretion of growth hormone. Children with VAD tend to secrete the hormone less than children who do not have VAD^(44)^. In addition, the effects of vitamin A on growth appear to be mediated by morbidity, in which VAD may lead to stunting in children with recurrent infections^(45)^.

Also, among the factors related to the child, not being breastfed was associated with the outcome (VAD) after multivariate analysis. It is known that the concentration of certain micronutrients in breast milk is closely related to maternal dietary intake and maternal plasma retinol concentration, which is an important source of vitamin A for breast-feeding infants^(46)^. Thus, breast-feeding can contribute considerably to meeting vitamin A intake recommendations during complementary feeding and in the early years of childhood^(47,48)^.

Some limitations need to be considered when interpreting the current study’s results. For example, its transversal nature precludes any causal inference. However, it suggests associations. The convenience sample limits the generalisability of results to general population. The study included a sample of children of low socio-economic status who reside in municipalities of high social vulnerability, thus limiting generalisation of the results to other strata, even within the South Region. The sample was selected through the PCU teams, which favours the selection of people who use this service over those who do not. However, primary care is the main form of health care and follow-up for children in these communities and the study’s announcement strategies were not limited to those who used these health services, which increases the probability of selecting a child population that is representative of these municipalities. The plasma retinol concentrations were not adjusted according to inflammation, and this could potentially have resulted in an overestimation of VAD^(17)^. However, even without adjusting for inflammation markers, the prevalence of VAD identified in the current study is not a public health problem^(1)^ in this sample of children. The prevalence of VAD found in the current study is lower than the prevalence of anaemia that served as the basis for the sample size calculation. Thus, it is possible that a larger sample would have been needed. The prevalence of anaemia data was for children under 5 years of age, which differs slightly from the age group considered in the present study. The coverage data of the NVASP in children of 6–11 months are not available, and we did not include children in this age group. This fact could underestimate the prevalence of VAD, considering that children aged 6–11 months could possibly be considered a risk group for this deficiency.

The study has some strengths such as an unprecedented sample from countryside poor municipalities in the South Region of Brazil, the contribution to guide the public policy of vitamin A supplementation and the use of the model as a hierarchical framework^(27)^ for the development of a multivariate analysis. The use of a single laboratory to quantify plasma retinol is also noteworthy, since it allowed for better control and standardisation of the technique.

Based on the prevalences found in the present study, the results suggest that VAD is not a public health problem in children aged 12–59 months in socio-economically vulnerable municipalities in the South Region of Brazil, suggesting a new panorama of this nutritional deficiency, even in regions of low socio-economic status in these three states. However, highly vulnerable children (those whose families were BFP beneficiaries, who had stunting and who were not being breastfed) were at higher risk for VAD, making it important to seek tracking strategies specifically focused on these populations. Public policies such as Brazilian Breastfeeding and Feeding Strategy in the Brazilian Health System (SUS Brazil)^(49)^ could be strengthened by ensuring the necessary intake of vitamin A and the adequate growth of children. Thus, in view of the current nutritional transition scenario, it is necessary to continuously monitor public policies, particularly the universal vitamin A supplementation programs in the municipalities studied. As future challenges, it is vitally important to investigate dietary practices to better understand the results found in order to provide appropriate food and nutrition programmes for children.
